# Uncharted territory: a narrative review of parental involvement in decision-making about late preterm and early term delivery

**DOI:** 10.1186/s12884-023-05845-6

**Published:** 2023-07-18

**Authors:** Frances J Mielewczyk, Elaine M Boyle

**Affiliations:** 1 Leicester City Football Club (LCFC) Research Programme, Department of Population Health Sciences, College of Life Sciences, George Davies Centre, University of Leicester, University Road, Leicester, LE1 7RH UK; 2 Department of Population Health Sciences, College of Life Sciences, George Davies Centre, University of Leicester, University Road, Leicester, LE1 7RH UK

**Keywords:** Decision-making, Parental involvement, Late preterm, Early term, Obstetric, Mode of delivery

## Abstract

Almost 30% of live births in England and Wales occur late preterm or early term (LPET) and are associated with increased risks of adverse health outcomes throughout the lifespan. However, very little is known about the decision-making processes concerning planned LPET births or the involvement of parents in these. This aim of this paper is to review the evidence on parental involvement in obstetric decision-making in general, to consider what can be extrapolated to decisions about LPET delivery, and to suggest directions for further research.

A comprehensive, narrative review of relevant literature was conducted using Medline, MIDIRS, PsycInfo and CINAHL databases. Appropriate search terms were combined with Boolean operators to ensure the following broad areas were included: obstetric decision-making, parental involvement, late preterm and early term birth, and mode of delivery.

This review suggests that parents’ preferences with respect to their inclusion in decision-making vary. Most mothers prefer sharing decision-making with their clinicians and up to half are dissatisfied with the extent of their involvement. Clinicians’ opinions on the limits of parental involvement, especially where the safety of mother or baby is potentially compromised, are highly influential in the obstetric decision-making process. Other important factors include contextual factors (such as the nature of the issue under discussion and the presence or absence of relevant medical indications for a requested intervention), demographic and other individual characteristics (such as ethnicity and parity), the quality of communication; and the information provided to parents.

This review highlights the overarching need to explore how decisions about potential LPET delivery may be reached in order to maximise the satisfaction of mothers and fathers with their involvement in the decision-making process whilst simultaneously enabling clinicians both to minimise the number of LPET births and to optimise the wellbeing of women and babies.

## Background

Compared to full-term infants, those born late preterm (34^+ 0^ to 36^+ 6^ weeks of gestation) and early term (37^+ 0^ to 38^+ 6^ weeks of gestation) are at increased risk of important neonatal morbidities [1–12] and ongoing health issues during childhood [13–40], adolescence and adulthood [23, 27, 34, 41–45]. Many late preterm and early term (LPET) births occur spontaneously, but others are planned in advance. Some of these reflect decisions in which the risks of continuing with pregnancies compromised by conditions such as pre-eclampsia, poor fetal growth, placental problems or infection have been judged to outweigh those of early delivery [46–49]; others are carried out in the absence of any clear medical indication [49–51].

Increasing awareness of the potential adverse consequences of LPET delivery, together with rising rates of LPET births during the late 20th and early 21st centuries [52–55], led to the development of various policies and initiatives aimed at minimising planned deliveries at these gestations. However, despite some significant reductions [49, 56–64], the number of LPET births remains high. In 2021, almost 33,700 late preterm and more than 151,500 early term births were recorded in England and Wales [65]. Taken together, these figures represent 30% of all live births. In addition to the implications for families, even modest increases in ill-health or reductions in intellectual ability across such large numbers place substantial burdens on healthcare services and resources [66].

Further reductions in planned LPET births would therefore be of significant benefit to individuals, families and the healthcare system. However, very little is yet known either about the decision-making processes involved in planning LPET deliveries, whether medically indicated or not, or about the involvement of parents in these. The aims of this paper are therefore to review the current evidence on the nature and extent of parental involvement in obstetric decision-making in general, to consider what can be extrapolated from this body of knowledge to decisions about LPET delivery, and to suggest avenues for further research into this complex and important issue.

## Methods

A lack of studies directly exploring parental involvement in decisions about LPET birth (i.e. birth between 34^+ 0^-38^+ 6^ weeks of gestation) precluded a systematic review of research on this topic. Instead, a comprehensive, narrative review of related literature was conducted by means of searches of relevant databases: CINAHL (The Cumulative Index to Nursing and Allied Health Literature), Medline, MIDIRS (The Maternity and Infant Care database), PsycInfo and PubMed. In view of the paucity of literature on this subject, a broad and inclusive search strategy was adopted, encompassing papers relating to humans and written in the English language, from the earliest date covered by each database to 18 August 2021. The following were accepted for inclusion: original research studies, analyses of secondary data (including data located online), clinical reports, review articles, commentaries, opinion papers, and proposals for new frameworks, guidelines and recommendations. There were no restrictions on time period or geographical location. The terms used in the search (combined with appropriate Boolean operators) are provided in Table 1.

**Table 1:** Terms used in the literature search

Topic area	Terms^1^
Gestation	Late preterm, early term, near term
Delivery	Medically indicated delivery, (elective) Caesarean section, (elective) induction of labour, abdominal delivery
Relationship	Parent, mother, father, maternal, paternal, family
Decision-making	Obstetric decision, decision-making, (parental) involvement

^1^ truncations and alternative spellings (including upper/lower case, hyphens and spaces) were added as appropriate, e.g. labo*, late pre-term; Cesarian Section

1,086 results were elicited by the searches. After preliminary scanning of titles and abstracts and the removal of duplicates, 53 papers were retained for further consideration. Eight papers were excluded after full text review. A further three with very early publication dates were also excluded, as their results were in line with those of more recent studies. The findings of the remaining 42 papers are included here. Summary details of these papers can be found in Table 2.

**Table 2:** Summary details of studies included in the review

Authors	Design of study/Type of paper	Sample size	Study time period	Country/countries of origin
Barker, Dunn, Moore et al. (2019)	Descriptive interview study	25	Sept - Dec 2015	Canada
Batton (2009)	Clinical report	N/A	N/A	USA
Berger, Bernet, El Alama et al. (2011)	Review article & revision of national guidelines	N/A	N/A	Switzerland
Blackwell (2011)	Review article	N/A	N/A	USA
Bylund (2005)	Analysis of unsolicited online birth stories	551	N/A	N/A
Chappell, Brocklehurst, Green et al. (2019)	Randomised control trial (RCT)	901	29 Sept 2014 - 10 Dec 2018	UK
Chen, Hutchinson, Nagle et al. (2018)	Observational & interview study	30^a^	Not stated	Taiwan
Cheng, McGough & Tucker Edmonds (2019)	Narrative review	N/A	N/A	USA
Chescheir & Menard (2012)	Review article	N/A	N/A	USA
Chhabra, Joymon, Lee et al. (2014)	Quantitative survey study	233	Mar 2008 - Sept 2010	USA
Coates, Donnolley, Foureur et al. (2021)	Quantitative survey study	340	Nov 2018 - July 2019	Australia
D’Souza, Shah & Sander (2018)	Commentary	N/A	N/A	Canada
Dageville, Bétrémieux, Gold et al. (2011)	Development of revised recommendations	N/A	N/A	France
Danerek, Maršál, Cuttini et al. (2011)	Quantitative questionnaire design	259	Mar 2007 - June 2008	Sweden
Dietz & Exton (2016)	Opinion paper	N/A	N/A	Australia & New Zealand
Dugas, Shorten, Dubé et al. (2012)	Systematic review and meta-analysis of RCTs	10^b^	1994 - 2010	Canada
Dupont, Blanc-Petitjean, Cortet et al. (2020)	Prospective population-based cohort study	1453	17 Nov 2015 - 20 Dec 2015	France
Edmonds, McKenzie, Hendrix et al. (2015)	Quantitative survey study	205	05 - 08 May 2013	USA
Eide & Bærøe (2020)	Development of new framework	N/A	N/A	Norway
Garel, Seguret, Kaminski et al. (2004)	Qualitative interview study	47^c^	May 1999 - March 2000	France
Guertzen, van Heijst, Draaisma et al. (2019)	Development of new nationwide framework	N/A	N/A	Netherlands
Gyamfi-Bannerman (2012)	Description of epidemiology and management	N/A	N/A	USA
Habiba, Kaminski, da Frè et al. (2006)	Cluster sampling cross-sectional survey	105 & 1530^d^	2001 - 2002	8 European countries^e^
Henderson, Gao & Redshaw (2013)	Secondary analysis of survey data	24,319	2010	UK
Hilder, Stubbe, Macdonald et al. (2020)	Observation & interview study	33^f^	June - Nov 2016	New Zealand
Longworth, Furber & Kirk (2015)	Narrative review	27	1992 - 2003	UK
Martin (2003)	Qualitative interview study	26	1997 - 1998	USA
Mathews, MacDorman, Thoma (2015)	Infant mortality statistics	N/A	2013	USA
Matthias (2010)	Observation & interview study	4^ g^	12 months (year not stated)	USA
Molkenboer, Debie, Roumen et al. (2008)	Quantitative questionnaire study	203	20 July 1998 - 21 April 2000	Netherlands
Morfaw, Gao, Moore et al. (2021)	Online survey	153	Aug - Sept 2019	Canada
Muggli, de Geyter & Reiter-Theil (2019)	Systematic review & qualitative synthesis	14	1996 - 2016	Switzerland
Munro, Kornelson, Corbett et al. (2017)	Qualitative interview study	35	April - Aug 2015	Canada
National Institute for Health and Care Excellence (NICE) (2021a)	National guidelines	N/A	N/A	UK
National Institute for Health and Care Excellence (NICE) (2021b)	National guidelines	N/A	N/A	UK
Nicholson, Kellar, Ahmad et al. (2016)	Sequential ecological dataset study	46	2007 - 2013	USA
Pirjani, Afrakhteh, Sepidarkish (2018)	Prospective cohort study	2086	Apr 2013 - Apr 2015	Iran
Redshaw & Henderson (2018)	Secondary analysis of population-based survey data	249	2012 - 2013	UK
Rikhardsdottir, Hardardottir & Thorkelsson (2019)	Retrospective cohort study	3411	01 Jan 1998 - 31 Dec 2017	Iceland
Tucker Edmonds, Savage, Kimura et al. (2019)	Qualitative interview study	54^ h^	April 2005 - Sept 2008	USA
White & Newnham (2019)	Review article	N/A	N/A	USA
Wilmink, Hukkelhoven, Lunshof et al. (2010)	Retrospective analysis of a national registry	20,973	01 Jan 2000 - 31 Dec 2006	Netherlands

^a^ 21 women and 9 obstetricians

^b^ 10 RCTs met the inclusion criteria

^c^ 17 obstetricians and 30 midwives

^d^ Neonatal intensive care units and obstetricians, respectively

^e^ France, Germany, Italy, Luxembourg, Netherlands, Spain, Sweden and UK

^f^ 20 patients and 13 obstetricians

^g^ 2 mother-obstetrician and 2 mother-midwife pairs

^h^ 40 pregnant women and 14 partners

## Literature review

### Parental involvement in obstetric decision-making

Numerous decisions are taken about treatment and care during pregnancy, labour and the neonatal period, with the aim of finding reliable, evidence-based and ethically justified solutions that are in the best interests of women and their babies [67, 68]. The right of parents to be involved in the making of such decisions has become widely recognised [67–76], including in recent national guidelines and recommendations in the UK and the Netherlands [72, 75, 76], and some form of discussion and/or negotiation is now common [77–83]. Few studies have reported the extent to which either mothers or fathers wish to be included in the decision-making process but there is evidence that both do want to be involved [77, 78, 82–87]. The literature concerning fathers is particularly scant but it has been noted that some have had a strong influence in relation to certain key decisions, such as when their partners should go to hospital during labour and the use and timing of epidural pain relief [87]. Others, however, have felt excluded from the process [86], with a lack of relevant knowledge and poor communication with health care professionals having been identified as barriers to their involvement [84]. Mothers vary in the extent of their desire to be included [84] but almost two-thirds have been found to prefer sharing decision-making with their clinicians and up to 51% consider their involvement in the decision-making process to have been too little [78, 82, 83, 85]. Since feeling insufficiently included has been identified as an independent determinant of dissatisfaction with care (albeit only, to date, in nulliparous women undergoing induction of labour) [85] and as many as 39% of decisions made during labour alone may be taken solely by clinicians [77], this is clearly an important issue both for parents and for those involved in the provision of maternity care.

Shared decision-making has been described as a collaborative process in which patients and healthcare professionals work together in order to reach a joint decision [75]. In practice, however, the relative power of the views and preferences of those involved is often unequal, with the balance being particularly influenced by clinicians’ opinions about the limits of parental involvement and contextual issues such as the nature of the issue(s) under discussion. Demographic and other individual characteristics have also been identified as important, as have the quality of communication and the information provided. These issues are discussed in turn below.

#### The opinions of clinicians

Clinicians’ views on the balance between the right of parents to have their preferences taken into account versus their own responsibility for the welfare of mother and baby are of central importance to the extent of parental involvement in obstetric decision-making: i.e. does the clinician see their role as being confined to the communication of information and the presentation of recommendations or do they consider it also to encompass the final authority over decisions? The most commonly reported opinion is that although parents’ views, preferences and authority are important there are limits beyond which these cannot be respected, particularly when preferences are judged to be unreasonable and/or likely to risk the wellbeing of either mother or baby [67–70, 73]. Some research has reflected this, showing clear differences in the extent to which parents have been involved in final decisions compared with prior discussions. In one study, for example, while large majorities of the parents of extremely preterm, very preterm and late-to-moderate preterm babies were involved in antenatal discussions about mode of delivery (62%, 73% and 77%, respectively) only small proportions of these were then allowed the final choice over how their babies were born (21%, 23% and 36%) [79]. Another investigation, concerning parents who had taken part in discussions about the anticipated birth of periviable infants, found that 22% had subsequently been excluded from decisions about mode of delivery and 22% from those relating to life support [82].

Other research has shown some parents to have been denied any opportunity at all to participate in the decision-making process. In one example, 26% of mothers whose babies had died after having had their life-support withdrawn were not involved to any extent in the decision that resulted in the withdrawal [81]; in another, 19% of women who underwent repeat caesarean sections did so because their obstetrician arranged the surgery without either having provided them with any information about the alternative or having sought their opinion [78].

Even in cases where women are granted the final authority over decisions, clinicians can still have a powerful effect on their choices – as has been shown in studies concerning planning for mode of delivery. 92% of the women in one such investigation either agreed or strongly agreed that their doctor or midwife knew what was best for them [83] and, in a study of mothers who had had previous caesarean sections, 62% of those whose clinicians provided them with both a recommendation and a clear explanation in connection with a subsequent delivery decided in favour of the recommendation [78]. However it is notable that, in the latter investigation, differences in the explanations provided by obstetricians of greater and lesser experience were associated with differences in women’s attitudes: a focus by less experienced obstetricians on detailing the potential for uterine rupture was associated with a greater reluctance to undergo VBAC.

However, while findings such as these demonstrate the potential influence of clinicians, they are not indicative of universal, unquestioning assent on the part of parents: the contentious nature of the issues underlying many obstetric decisions makes it common for disagreements to arise. This is reflected in the rise in importance of Ethics Consultations (ECs) in both Europe and the United States. Aimed at supporting informed, considered decision-making, ECs comprise experienced healthcare professionals from a range of relevant disciplines and can be requested by parents as well as clinicians. However, a review of 32 EC decisions found only 12 (37.5%) to have supported the wishes of the parents [80]. In the remaining cases, these were judged as either not reflecting the best interests of the baby or as involving clinical approaches regarded as substandard. EC decisions are not binding, though, and two of those reviewed had subsequently been overruled by the head physician (one in favour of parents’ preferences and one against) – a finding that further emphasises the scope of clinicians’ authority.

Few investigations have considered similarities and differences in the views of different types of clinician but some qualitative studies have explored those of obstetricians and midwives. One such study found that, compared with obstetricians, midwives reported less involvement of parents of extremely preterm infants in decisions on both mode of delivery and postnatal resuscitation [88]. Another example found that midwives both demonstrated an acceptance of women having decision-making power and perceived it as part of their own role to provide them with the education needed to make properly informed choices while, by contrast, although the obstetricians expressed a willingness to engage in shared decision-making, in practice they imposed limits on maternal choice and retained the final authority [73].

Research has also suggested a number of other clinician-related influences on shared decision-making in an obstetric context. For example, an understanding of the process and how to apply it has been proposed as a facilitator [89] while the following have been identified as barriers: feeling overwhelmed when attempting to include parents in decisions where multiple alternative management strategies and potential outcomes exist [90]; fear of the stress and negative emotions that may be experienced by parents who are involved in decisions [89]; and seeing parental involvement within certain contexts as being unhelpful to parents [88]. Cultural factors, fear of litigation and national/organisational factors have also been shown to be influential [91].

#### Contextual influences

Both the opinions held about parental involvement in obstetric decision-making and the extent to which parents are actually included have been shown to vary according to contextual influences such as the nature of the issue under discussion. In particular, support for parents’ right to choose is reduced in cases where there is no clear medical indication for a requested intervention or where the procedure itself is very risky. For example, 77% of midwives in one study thought obstetricians should not agree to a request for a caesarean section in the absence of any current medical indication unless the request arose from previous maternal or fetal complications [70]. However, high levels of parental involvement in decisions may be granted in circumstances where no benefit of medical intervention would be anticipated: for example, the American Academy of Pediatrics has advised that, in cases where a positive outcome is considered very unlikely regardless of intervention, parents should be given the opportunity to decide whether or not resuscitation should be initiated [92].

Planned mode of delivery is another relevant contextual issue: less positive decision-making experiences have been reported by women planning vaginal delivery compared to those planning caesarean section, while the latter have themselves felt less involved in decisions than those planning induction of labour [78, 83, 93]. Conversely, though, in a study of women who had experienced a previous caesarean birth, all those who opted for a subsequent vaginal delivery felt involved in that decision while almost a fifth of those who underwent repeat caesarean sections reported being excluded completely from the decision-making process [78].

Little research has so far specifically addressed the issue of parental involvement in decisions concerning medication, and its results have been mixed. While one study found mothers to have been involved to a greater extent in decisions about pain medication than in those about any other aspect of the birthing process [77], another showed patient preference to have had a weaker influence in relation to steroid administration than to other aspects of management. It was concluded that this latter finding was due to the relative strength of the evidence-base supporting the use of steroids in the context concerned – the management of periviable deliveries – compared to those relating to the other issues under consideration [94]. This finding highlights the importance of taking the strength of relevant evidence into account when considering influences on shared decision-making in specific contexts.

#### Demographic and other individual characteristics

While the association of demographic and other individual characteristics with parental involvement in decision-making is another under-researched area, some related differences have been identified. For example, less positive experiences have been reported by women from ethnic minorities and diverse cultural backgrounds [83, 95] with one study having found that, compared to White women, those from minority ethnic groups were less likely either to be spoken to in ways they could understand or to feel sufficiently involved in decisions throughout their maternity care [95]. In addition, large differences have been found across European countries in both obstetricians’ attitudes towards maternal autonomy and their willingness to comply with maternal requests for non-medically indicated caesarean section, which ranged from 15 to 79%. Guidelines on the handling of such requests also varied between countries [68].

In other studies, less positive decision-making experiences were reported by primiparous women and those who did not receive continuity of maternity care [83]. Consistent with the findings reported earlier about differences in the opinions of midwives and obstetricians, the extent of women’s involvement in birth-related decisions (as recounted in online birth stories) was greater for those with midwives as their clinicians than for those whose clinicians were physicians [77].

#### Communication and information

Numerous researchers have made the case that, for shared decision-making to be effective, it is essential that comprehensive, evidence-based information about all available options, including their associated risks and benefits, be clearly communicated to parents [67, 68, 71, 75, 96]. This has been exemplified in a study of parents invited to take part in shared antenatal decision-making consultations, after which they rated the communication that had taken place in very positive terms, reporting their questions to have been answered, their feelings heard and their anxieties acknowledged [97]. Positive experiences such as these have not always been matched, however, with women attending antenatal clinics across the breadth of one country being described by midwives as having been neither sufficiently listened to nor adequately informed [88]. In other examples, 40% of women whose babies had been delivered by caesarean section were unaware of the risks associated with that mode of delivery [79], while 30% of mothers did not know that delivery by caesarean section is not safer than vaginal delivery and 33% were unaware that elective late preterm birth is not advisable [98]. Of course, it is possible that the information in this last study had been communicated to but not assimilated by the women concerned. Evidence has shown assimilation to be best achieved when information is presented both orally and in writing, either computer-based or on paper: a combination noted to be preferred by most parents [71, 79]. However, written information is less commonly provided than oral and the extent of its provision does not always meet that which is desired [79]. A range of other decision aids have also been found effective in increasing the assimilation of knowledge, including decision analysis tools, decision trees and both individual and group counselling [71].

In other investigations of the components of effective communication, it has been suggested that information should be presented in a sensitive and supportive manner [67] with clinical risks being explicitly addressed [97], and that both ongoing dialogue between all relevant parties and sufficient time for deliberation and evaluation of the information provided are required [67, 68, 75, 78]. The timing of discussions may also be important, as reflected in the proposal that, in order to prevent uninformed decisions being made early in a subsequent pregnancy, discussions should take place soon after a caesarean birth of the reasons for that mode of delivery and the likely options regarding future births [74].

Lastly, it has been argued that certain situations, such as requests for caesarean section, require discussions to be tailored to the specific needs of the individual: since such requests may be influenced by a complex combination of perceptions, values, past experiences, motivations and fears, it has been recommended that each case be addressed on an individual basis, with a greater emphasis being placed by clinicians on understanding what underlies the request before providing relevant, accurate information and a well-justified medical recommendation [68, 91].

### Parental involvement in decisions specific to LPET delivery

Apart from investigations into the effectiveness of programmes aimed at reducing planned deliveries before 39 weeks of gestation, research specific to decisions on LPET birth is sparse. However, some results have been reported and a certain amount can also be learned or inferred from consideration of the factors already highlighted as contributing to obstetric decision-making as a whole.

The central issue of the safety of babies is clearly of equal importance across the gestational spectrum. In the case of LPET delivery, however, there is the additional need for the risk of the adverse outcomes known to be associated with birth at these stages to be balanced against a possible increase in the risk of stillbirth at term. Findings in relation to this issue have so far been mixed, as shown by explorations of the impact of the “39-week” rule, introduced in the US in 2009 with the aim of avoiding non-medically indicated delivery before that gestation. While early results suggested substantial associated rises in the rate of stillbirth [99] more recent studies and figures have found no evidence of any effect of the policy on either stillbirths or any other adverse perinatal outcome [63, 100]. However, the inadequacy of the tools currently available to distinguish cases where life could be saved by early delivery from those where birth could safely be deferred [49] makes it inevitable that the opinions and judgements of clinicians will exert a powerful influence over decisions about the timing of delivery. This is highlighted by recommendations that refer to the need for decisions to be weighed up by clinicians [101] and based on the best available evidence [102] while omitting any reference to possible parental input. Further illustration comes from a review of 790 caesarean sections performed at early term, in which it was argued that almost two-thirds could have been postponed until 39 weeks of gestation: since 50 of the cases included in that group (6.3% of the total) had been performed in accordance with maternal requests, those requests were clearly judged by the reviewers as dismissible [51].

Contextual issues such as complications of pregnancy will also have a bearing on parental involvement in decisions specific to planned LPET delivery. In one study, for example, it was recommended that women with late pre-term pre-eclampsia be given the opportunity to take part in discussions of the relative benefits and risks of planned early delivery versus expectant management in their specific case [103]. By contrast, however, decisions concerning the timing of delivery in cases of stable placenta praevia (where deterioration may occur rapidly and without warning) have been presented as resting solely with the obstetrician [104].

Finally, as up to 60% of all caesarean sections are carried out before 39 weeks of gestation [105, 106], the issues previously discussed regarding parental involvement in mode of delivery planning are also relevant to LPET birth: differences in the extent of involvement according to the mode of delivery being planned and the presence or absence of relevant medical indications; the influence of clinicians’ opinions and recommendations; differences according to demographic factors; communication and information-related issues; and the impact of past experiences, motivations and values.

## Discussion

### Summary of findings

This review has provided an overview of current knowledge about parental involvement in obstetric decision-making, particularly in relation to the views and expectations of parents and healthcare professionals, and factors that may influence the nature and extent of the contributions parents are able to make. It has highlighted the importance of clinician-related factors, most notably their opinions on the limits of parents’ rights when the wellbeing and safety of the mother and/or baby is at risk. It has identified some important contextual influences on parental involvement, including the potential mode of delivery and the strength of the evidence-base concerning the issue(s) under discussion. Demographic factors, such as women’s ethnic and cultural backgrounds, as well as the nature of the information provided to parents, the mode of its presentation and the manner in which it is delivered have all also been shown to have a bearing.

Many of these findings have direct relevance for parental involvement in decision-making about potential LPET birth. Of particular salience, in relation to safety and wellbeing, are clinicians’ opinions about the relative risks of term stillbirth and LPET birth in any individual case. The nature of presenting complications of pregnancy and preferences around mode of delivery are also influential contributors to the nature and extent of parental involvement in the decision-making process.

### Strengths and limitations of the review

This review addresses a topic about which there is limited published literature. However, 42 papers were identified that usefully contribute to knowledge and understanding of the issues surrounding parental involvement in obstetric decision-making in general and, more specifically, in discussions and decisions about possible planned LPET birth. Although some publications were up to 20 years old, it was considered appropriate to include them if they contributed information that was that was considered still useful and not duplicated by later publications. Over 80% of included papers were published after 2010 and almost 50% between 2017 and 2023.

The paucity of prior research on the topic made it unsuitable for systematic review. Instead, a narrative review was undertaken with the aim of appraising the extent of current knowledge and identifying areas for future research. The narrative approach has some acknowledged limitations. Primary among these is subjectivity in study selection, leading to potential biases in reporting [107]. In this review, while both authors identified terms to be used in searching for literature and agreed the inclusion criteria, the first author was solely responsible for decisions about eligibility of papers for inclusion, making subjectivity at this stage a potential issue. However, as detailed in Table 2, the papers included in the review display a breadth that suggests any bias in selection was kept to a minimum: the papers were of a variety of types, with those reporting original research being supplemented by reviews, guidelines, commentaries and a clinical report; the studies adopted a range of quantitative and qualitative designs; and they originated in 17 countries across the UK and Europe, North America, Australasia, Scandinavia and Asia.

### Directions for future research

This review has highlighted one crucial overarching question: how can decisions regarding planned LPET delivery be reached in such a way as to maximise the satisfaction of mothers and fathers with their involvement in the decision-making process (and thereby possibly also with their maternity care overall) whilst simultaneously ensuring that clinicians are able both to keep the number of births at this gestation to a minimum and to discharge their responsibility to optimise the wellbeing of women and babies? If this question is to be answered, considerable further investigation is required.

A greater understanding is needed of the preferences of mothers and fathers regarding their inclusion in decision-making, the extent to which their experiences align with their preferences, and the consequences of any lack of alignment on their overall satisfaction with the maternity care received. Ascertaining the nature and extent of any discrepancies between the views of parents, obstetricians and midwives on parental input to decision-making about LPET birth could inform the nature and timing of the information and support offered to parents. This could improve parental satisfaction with the decision-making process and possibly also with their maternity care overall.

Differences in levels of parental involvement according to demographic and other relevant individual characteristics also need further exploration, including any impact of similarities or differences in such factors between clinicians and parents. Also, given the reported differences in levels of parental involvement in preliminary discussions compared with final decisions, it would be useful to determine clinicians’ interpretations of what comprises fully shared decision-making and to further explore potential facilitators and barriers to their achieving recommended levels of sharing. Increased awareness of clinicians’ opinions specific to planned LPET is also required, both in general and in relation to contextual factors such as mode of delivery (including where non-medically indicated) and the strength of any associated evidence base. Further investigation of the nature and quality of effective communication of information relevant to LPET delivery could also enhance our understanding of the impact of these on parental involvement and satisfaction.

As would be expected from research carried out in connection with an only recently identified issue of importance, most investigations have so far addressed only the broader questions relevant to this topic. It is now necessary to start exploring in some depth the complexity of influences on decision-making processes. With respect to parents, for example, little attention has so far been paid to the impact of their existing beliefs about the process of childbirth, fears surrounding the possible adverse outcomes of vaginal delivery of a full-term baby, or their educational level and skills in written and spoken English (whether this is their first or a subsequent language). Parental participation in decision-making in the context of known mental health issues and/or domestic violence also merits exploration, as these circumstances may be associated with other factors indicating an increased risk of LPET birth, such as stress and the use of antidepressant medication.

The potential contribution of clinician-related factors have also received little attention, with issues such as fear of litigation and a desire to avoid eliciting negative emotions in parents having been mentioned only briefly and others, such as the outcomes of relevant past decisions and their own, personal obstetric experiences, having not been addressed at all. Given the burden placed by the large number of LPET births on healthcare resources, it would also be helpful for health economic analyses to be included in future evaluations of the appropriateness of LPET decisions.

## Conclusions

The extent to which parents can or should be involved in decision-making about possible LPET delivery is a matter of some contention. While there is widespread recognition of the value of shared decision-making, a broad range of influences may shape the balance of power within the decision-making context. Only by considering the full breadth of possible influences and their relative contributions to the decision-making process, can a more fully informed understanding of how a balance between the preferences of parents and the responsibilities of clinicians be achieved.

## Data Availability

Not applicable.

## List of abbreviations

EC: Ethics consultation
CINAHL: The Cumulative Index to Nursing and Allied Health Literature
LPET: Late preterm and early term
MIDIRS: The Maternity and Infant Care database
